# Complications of Severe Odontogenic Infections: A Review

**DOI:** 10.3390/biology11121784

**Published:** 2022-12-08

**Authors:** Timothy W. Neal, Thomas Schlieve

**Affiliations:** Department of Surgery, Division of Oral and Maxillofacial Surgery, University of Texas Southwestern Medical Center, Dallas, TX 75390, USA

**Keywords:** infection, odontogenic, abscess

## Abstract

**Simple Summary:**

Odontogenic infections are most commonly caused by dental caries. Localized infections may be treated simply, while severe odontogenic infections that have spread into the spaces of the head and neck require hospital admission and operating room treatment. The majority of severe odontogenic infections are treated routinely, however, there are several complications that may arise. Patient risk factors, diagnostic tools, and clinical features of the complications associated with severe odontogenic infections are described.

**Abstract:**

Severe odontogenic infections are routinely treated with little associated morbidity and mortality. Improvements in surgical techniques, antibiotic treatments, and imaging modalities have made associated complications exceedingly rare. A number of complications have been described in the literature including airway obstruction, descending necrotizing mediastinitis, orbital abscess, septic cavernous sinus thrombosis, cerebral abscess, sepsis, necrotizing fasciitis, and Lemierre’s syndrome. The purpose of this article is to discuss the pathophysiology of severe odontogenic infections and the risk factors associated with the development of complications. Given the morbidity and mortality of these conditions, it is important to review the clinical features of each and the diagnostic tools that aid in early recognition.

## 1. Introduction

Dental pain is a common reason for emergency room presentation. Of all emergency room visits in the United States, about 4.3% are dental related [1]. Many hospital emergency rooms are not equipped to provide appropriate restorative and preventative dental care. While outpatient preventative treatment is the most cost-effective way to manage dental complaints, these services may be cost prohibitive to many. When patients present to the emergency room with dental related complaints, 90% leave with prescription analgesics and antibiotics instead of source control [2]. Dental related emergency department visits are a source of overcrowding in a system already overburdened by nonurgent or preventable health conditions. In one year, the estimated cost associated with emergency department visits in the United States for dental caries was $110 million dollars [3].

When dental caries are not addressed, they may progress to odontogenic infections. These infections may be localized or disseminated. Localized infections may be treated at bedside in the emergency department or in the outpatient setting with source control and simple incision and drainage. Once disseminated into the fascial spaces of the head and neck, these infections are categorized as severe odontogenic infections and require incision and drainage in the operating room setting. The mortality rate associated with severe odontogenic infections has significantly declined with the advent of antibiotics and improved surgical technique. The vast majority of odontogenic infections, localized or severe, are routinely managed with little morbidity or mortality. However, there are a number of complications associated with severe odontogenic infections that are worthy of review, including airway obstruction, descending necrotizing mediastinitis, orbital abscess, septic cavernous sinus thrombosis, cerebral abscess, sepsis, necrotizing fasciitis, and Lemierre’s syndrome [4,5,6]. 

### 1.1. Pathophysiology, Patient Factors, and Microbiology 

Odontogenic infections are unique in that oral cavity bacteria have a passageway deep into the head and neck by way of the teeth and surrounding structures. The infection usually progresses through three phases: inoculation, cellulitis, and abscess formation. When bacteria gain access to the pulp chamber, the infection may spread to the tooth apex. At the apex of the tooth, the anatomical location determines the pathway of dissemination (Table 1). In the case of mandibular molars, apical infection that perforates the medial cortex of the mandible may spread to the sublingual space if the area of perforation is superior to the mylohyoid muscle. If the area of perforation is located inferior to the mylohyoid muscle, the infection will disseminate to the submandibular space. In cases where the infection perforates the lateral border of the mandible, this may lead to either vestibular space or buccal space infections depending on the attachment of the buccinator muscle by the same principal. Infections involving a single space may progress through anatomical communications. For example, a submandibular space infection may disseminate posteriorly to involve the pterygomandibular space. Additionally, infection associated with a mandibular third molar may disseminate directly to the pterygomandibular space, the lateral pharyngeal space, to the retropharyngeal space, and into the danger space to the mediastinum. Mandibular molars are the most frequent sources of severe odontogenic infections. In a prospective study by Flynn et al. investigating severe odontogenic infections, 68% were caused by mandibular third molars [7]. In single space infections the most common space involved is the buccal space, as compared to multiple space infections where the most common space involved is the submandibular space. 

**Figure 1:**
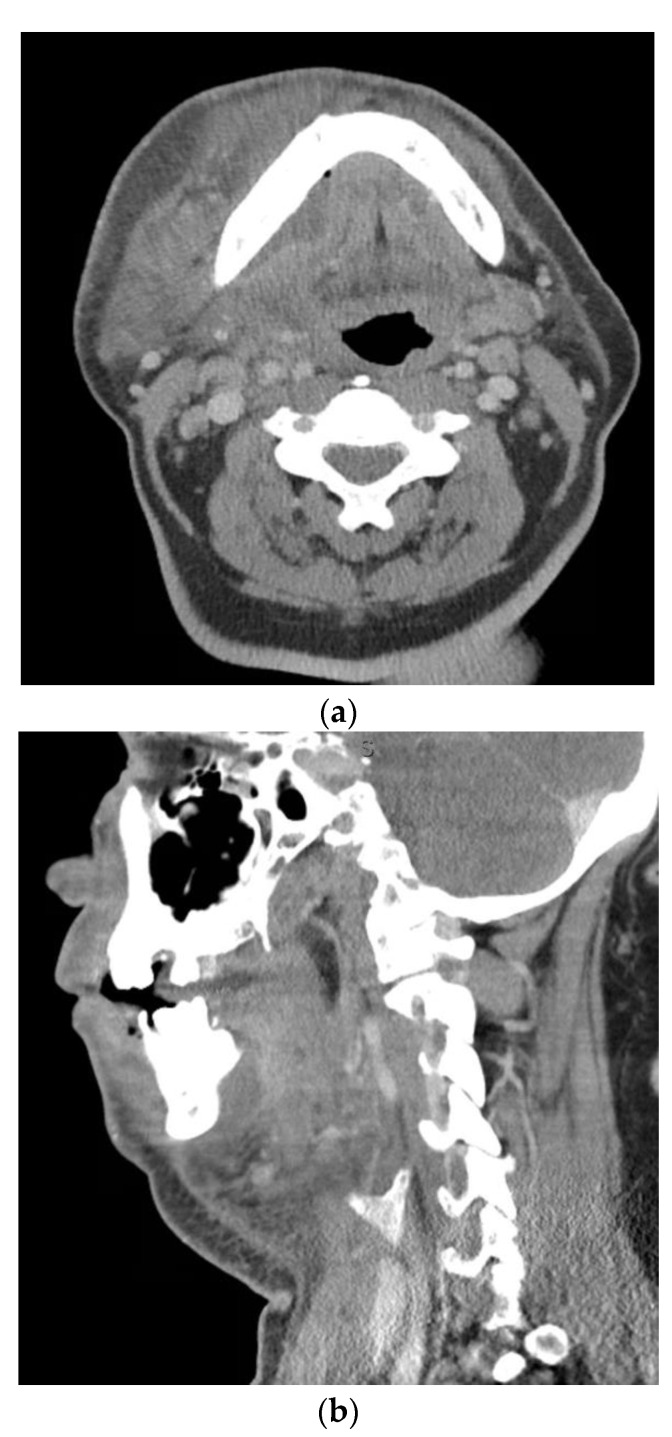
Axial (**a**) and sagittal (**b**) computed tomography scan views of patient with right submandibular, sublingual, and submental abscess with associated odontogenic severity score of six.

Odontogenic infections present along a spectrum of severity. A useful measure both clinically and academically is the odontogenic infection severity score developed by Flynn et al. [8]. This scoring system provides valuable insight into the extent of the infection based on the risk to the airway. Infections involving low risk spaces (vestibular, subperiosteal, infraorbital, buccal space) are assigned a score of one. Infections involving medium risk spaces (submandibular, submental, sublingual, pterygomandibular, submasseteric, superficial temporal, deep temporal) are assigned a score of two. Infections of high-risk spaces (lateral pharyngeal, retropharyngeal, pretracheal, danger space, mediastinum, intracranial space) are assigned a score of three. The odontogenic infection severity score is the sum of each score assigned to each fascial space involved at presentation. As an example, Figure 1 presents a patient with an odontogenic infection severity score of 6, given the involvement of the right submandibular, sublingual, and submental spaces. Previous studies have shown that when patients present with odontogenic infection severity scores of greater than or equal to five, there are increased associated hospital costs and consumption of postoperative as-needed opioid medications [9,10].

Certain patient specific factors may create an environment that allows the dissemination of odontogenic infections. Any condition that causes immunosuppression may allow dissemination more rapidly. Perhaps the most common immunosuppressing condition is diabetes. In the United States alone, an estimated 37.3 million people have diabetes [11]. Diabetes causes significant microvascular and macrovascular changes. Additionally, there is impairment of cytokine production, leukocyte recruitment, and leukocyte function [12,13,14,15]. This is especially true for patients with poorly controlled diabetes, as the susceptibility to infection increases drastically [16,17]. In a study investigating severe odontogenic infections, Hammad et al. found a significantly higher blood glucose level at admission in patients with odontogenic infection severity scores greater than or equal to five [18]. Other reported factors that play a role in the development of severe odontogenic infections include obesity, drug abuse, tobacco abuse, alcohol abuse, and malnutrition [19,20,21].

Many studies have investigated the microbiology of odontogenic infections. A common theme among all of these studies is the diversity of microbes present. Odontogenic infections are generally polymicrobial and the majority are caused by mixed aerobic and anaerobic bacteria. In a study by Siqueria Jr et al., over 460 unique bacteria taxa belonging to 100 genera and 9 phyla were identified from culture and molecular studies. The same study also noted that fungi, archaea, and viruses contribute to the microbial diversity, although to a lesser degree [22]. In a study by Khemaleelakul et al., a total of 127 strains of bacteria were isolated from needle aspiration of odontogenic infections. Of the strains isolated, 63% were anaerobes while 37% were aerobes. In the 17 cases investigated, 82% of cases had strict anaerobes and microaerophiles as the dominant bacteria [23]. The diversity of microbes present in odontogenic infections reflects the diversity of the oral cavity microflora. Generally, the infection is initiated by aerobic bacteria. As the bacterial load increases, and the infection progresses, anaerobic bacteria dominate. Previous reports show that standard culturing methods of odontogenic infections yields on average 2–8 species. When molecular methods are used, the average is around 18 species [24,25]. For localized odontogenic infections, cultures are generally not needed as source control with dental extraction and incision and drainage allows for adequate coverage with a single antibiotic. However, in severe odontogenic infections culture and sensitivity testing is always warranted. This is also true for immunosuppressed patients, as atypical pathogens may be present. In terms of clinical relevance, the five most common species of bacteria found in severe odontogenic infections is displayed in Table 2. 

### 1.2. Airway Obstruction 

**Figure 2:**
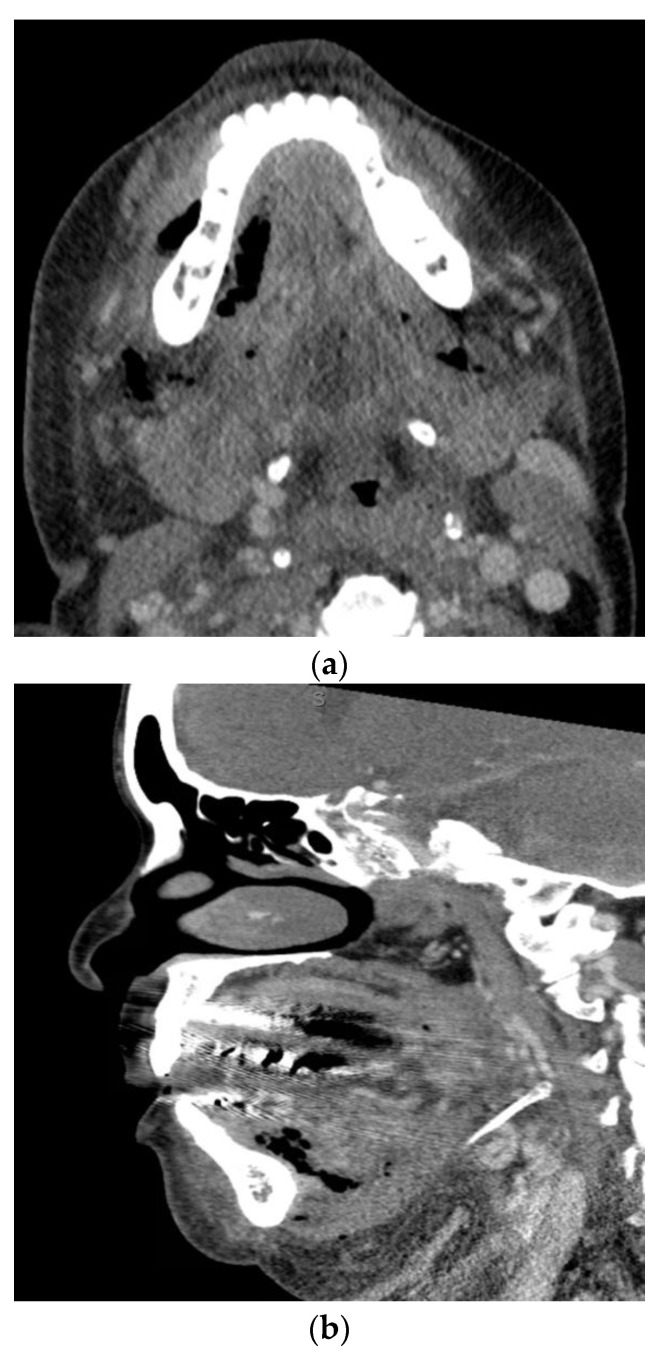
Axial (**a**) and sagittal (**b**) computed tomography scan views of a patient with Ludwig’s angina.

A feared complication of severe odontogenic infections is airway obstruction. When these infections involve deep fascial spaces, significant airway narrowing, and deviation may occur. Of particular importance is Ludwig’s angina which involves the submental space, the bilateral submandibular spaces, and the bilateral sublingual spaces Figure 2. The mandibular molars are the most common sources of infection leading to Ludwig’s angina. The clinical presentation includes pain, drooling, dysphonia, brawny neck edema, and tongue protrusion or elevation. The tongue is often pushed up and backward due to the fascial spaces involved, leading to airway obstruction. With improved imaging techniques, antibiotics, and a shift to early surgical treatment, the mortality rate from Ludwig’s angina has decreased from 50–54% to around 4–8% [26,27,28]. 

The symptoms of severe odontogenic infections include trismus, pain, swelling, dysphagia, dysphonia, and fever [29]. Many of these symptoms make securing the airway more challenging. Additionally, distortion of the anatomy and immobility of the tissues may create a difficult scenario for the provider attempting to establish a secure airway. In a study by Riekert et al. of 499 severe odontogenic infection patients, limited mouth opening was associated with difficult airway management. In the same study, four patients (0.8%) required tracheostomy [30]. Intubation methods such as flexible bronchoscopy and video-assisted laryngoscopy have improved the ability to establish an airway in patients with severe odontogenic infections. In a study by Schumann et al. of 100 patients with severe odontogenic infections, video-assisted laryngoscopy was successful in all patients while conventional tracheal intubation failed in 17 out of 50 patients [31]. Both flexible bronchoscopy and video-assisted laryngoscopy have high success rates and have certainly contributed to the decreased mortality seen in patients treated for Ludwig’s angina [32,33]. 

### 1.3. Descending Necrotizing Mediastinitis 

Severe odontogenic infections that spread to the deep neck may gain access to the mediastinum. When acute purulent mediastinitis from an odontogenic source occurs, it is termed descending necrotizing mediastinitis [34]. The diagnostic criteria, as defined by Estrera et al., includes clinical manifestations of severe infection, establishment of a relationship between an oropharyngeal or cervical infection and subsequent mediastinitis, demonstration of radiographic features characteristic of descending necrotizing mediastinitis, and documentation of a necrotizing mediastinal infection at the time of operative debridement [35]. When the source is odontogenic, the mandibular molars are the usual culprit and spread occurs from the retropharyngeal space to the danger space that is confluent with the mediastinum. This complication is incredibly rare, but serious, and life threatening when it occurs. Early studies of descending necrotizing mediastinitis reported a mortality rate of 52% [36]. While surgical techniques and antibiotics have improved with time, the mortality rate remains high at 25–40% [35,37]. This is owed to the aggressive nature of this form of mediastinitis as seen in a case report by Takao et al. The patient described was a 54-year-old that developed descending necrotizing mediastinitis after local retropharyngeal space abscess drainage and administration of systemic antibiotics [37]. 

Early recognition of this rare complication is of paramount importance. Improvements in imaging modalities has allowed for earlier detection, and improved survival. The clinical progression of descending necrotizing mediastinitis was classified by Endo et al. based on anatomical involvement. They defined type 1 as localized to the upper mediastinum above the tracheal bifurcation, type 2a extends to the lower anterior mediastinum, and type 2b extends to the anterior and lower part of the mediastinum [38]. In most cases the treatment is surgical with cervicotomy and thoracotomy in combination with systemic antibiotic coverage for aerobic and anaerobic bacteria. 

### 1.4. Orbital Cellulitis, Abscess, and Septic Cavernous Sinus Thrombosis 

It is rare for severe odontogenic infections to involve the orbit. However, when the orbit is involved, there is significant morbidity and mortality associated. The mortality rate associated with orbital cellulitis and orbital abscesses in the pre-antibiotic era was 17%, owed to intracranial complications. Vision loss was also common with an associated rate of 20%. With improvement in antibiotic treatment, the rate of vision loss has decreased to well below 10% [39,40]. It is important to note that around 80% of orbital cellulitis and abscess cases are caused by bacterial rhinosinusitis. The minority of cases are caused by trauma, infections of the face, middle ear, tonsils, or teeth [41,42]. Odontogenic infections usually gain access to the orbit through the maxillary sinus and associated facial vasculature. However, these infections may gain access posteriorly, as seen in a case report by Rothschild et al., where the odontogenic infection spread to the deep temporal space, pterygopalatine fossa, and into the orbit through the inferior orbital fissure [43]. 

Infectious complications of the orbit are classified anatomically by the orbital septum. The classifications defined by Chandler et al. include preseptal cellulitis or abscess, postseptal (orbital) cellulitis, subperiosteal abscess, orbital abscess, and cavernous sinus thrombosis [44]. Orbital cellulitis and abscess are further classified as extraconal or intraconal depending on the location in relation to the extraocular muscles [45]. 

The cavernous sinuses are one of the seven paired dural venous sinuses that drains venous blood from the cranial cavity. These sinuses are within the cranial cavity between the periosteal and meningeal layer of the dura mater. The paired cavernous sinuses communicate anteriorly and posteriorly and are located on either side of the sella turcica of the sphenoid bone [46]. Along the lateral surface of the cavernous sinuses are the oculomotor, trochlear, ophthalmic, and maxillary nerves. The internal carotid, sympathetic plexus, and abducens nerves are located medially in the cavernous sinuses. When the cavernous sinuses are involved, the local anatomy provides important clinical clues. Patients typically present with unilateral headache, tachycardia, hypotension, fever, eye pain, periorbital edema, chemosis, proptosis, ophthalmoplegia, and loss of vision [47,48,49,50]. 

Blood empties into the cavernous sinus from the superior and inferior ophthalmic veins, the sphenoparietal sinus, and the middle cerebral veins. The cavernous sinus drains into both the superior and inferior petrosal sinuses, and the emissary veins that lead to the pterygoid plexus. Because these venous connections are valveless, infection and thrombi can travel in anterograde or retrograde fashion [51]. It is important to note that cavernous sinus thrombosis can have aseptic causes, therefore cases that are septic in nature are termed septic cavernous sinus thrombosis. The mechanism is related to embolization of bacteria triggering thrombosis and infection within the cavernous sinus. The majority of septic cavernous sinus thrombosis cases are caused by sinusitis [52]. Severe odontogenic infections represent less than 10% of all septic cavernous sinus thrombosis cases [53]. To put the rarity of this condition into perspective, the incidence of cerebral venous sinus thrombosis is about 15.7 cases per million people per year. Of these cases, 1% are located in the cavernous sinuses [54]. Regardless, it is important to recognize and promptly treat this rare complication of severe odontogenic infections, given the morality and significant risk of vision loss.

### 1.5. Cerebral Abscess 

Severe odontogenic infections may spread intracranially through four theoretical routes (1) systemic bacteremia; (2) through the cavernous sinus by way of the facial and pterygoid venous systems; (3) inoculation via contiguous extension or by introduction of foreign objects; (4) lymphatic drainage [55]. Systemic bacteremia is thought to be the primary route of dissemination to the cranial vault. In a systematic review by Moazzam et al. of intracranial bacterial infections of odontogenic origin, 26.6% of the study sample had involvement of the ipsilateral frontal lobe or bacterial meningitis and 5% had clinical evidence of cavernous sinus involvement. Additionally, 13.3% of cases involved posterior intracranial structures, and there was no predilection for mandibular versus maxillary involvement. These results, as the authors stated, support the theory of hematogenous spread [55]. If the primary route of dissemination was through direct venous drainage, then involvement of the cavernous sinus and ipsilateral frontal lobe would be more frequent. Additionally, there would be a greater discrepancy in the anatomical location of the odontogenic source, in favor of the maxilla. In a more recent systematic review by Carvalho Correa Lisboa et al., the authors found that maxillary molars were more frequently involved than mandibular molars. The authors explain that this finding was likely due to the inclusion of more studies as compared to the systematic review by Moazzam et al. The study found that in 31.1% of cases the ipsilateral side of the brain affected coincided with the odontogenic infection side and the pterygoid plexus and cavernous sinus were involved in 0.7% of cases. While maxillary teeth were more frequently involved, these results still suggest that hematogenous spread is the predominate route [56]. Rarely, severe odontogenic infections may disseminate intracranially by direct extension. This can be seen in a case report by Sakamoto et al., where a 61-year-old male with left parapharyngeal and masticator space abscesses developed a temporal lobe abscess by direct extension through the foramen ovale [57].

Cerebral abscess formation from an odontogenic source is rare. The incidence of cerebral abscess is 1 case per 100,000 people per year and 2–5% are attributed to an odontogenic source [58,59]. The frontal lobe is most commonly affected, and when the source of the cerebral abscess is odontogenic, the most common bacterial species cultured are Streptococcus, Fusobacterium, and Porphyromonas [59]. Patients typically present with neurologic symptoms, fever, and headache. Three criteria were defined by Ewald et al. to confirm the odontogenic origin of a cerebral abscess (1) no other infectious source has been found; (2) microbiological spectrum should represent the oral microflora; (3) clinical and radiographical signs of an acute or chronic dental or paradental infection [60]. With improvement in diagnostic modalities, antibiotic treatments, and surgical techniques the mortality rate has decreased from 40% to 10% in the past 50 years [61]. 

### 1.6. Sepsis

Sepsis is defined as life-threatening organ dysfunction caused by a dysregulated host response to infection. Organ dysfunction is identified using the Sequential Organ Failure Assessment (SOFA) score. This score evaluates each organ system and assigns a value based on function. The components of the score include the partial pressure of oxygen in the arterial blood, platelets, bilirubin, mean arterial pressure, Glasgow Coma Scale score, creatinine, and urine output [62]. There is a direct relationship between the SOFA score and mortality [63]. Organ dysfunction can be identified as an acute change in total SOFA score of greater than or equal to two. When a hospital patient has a score of greater than or equal to two, there is an associated 10% overall mortality risk [62]. 

**Figure 3:**
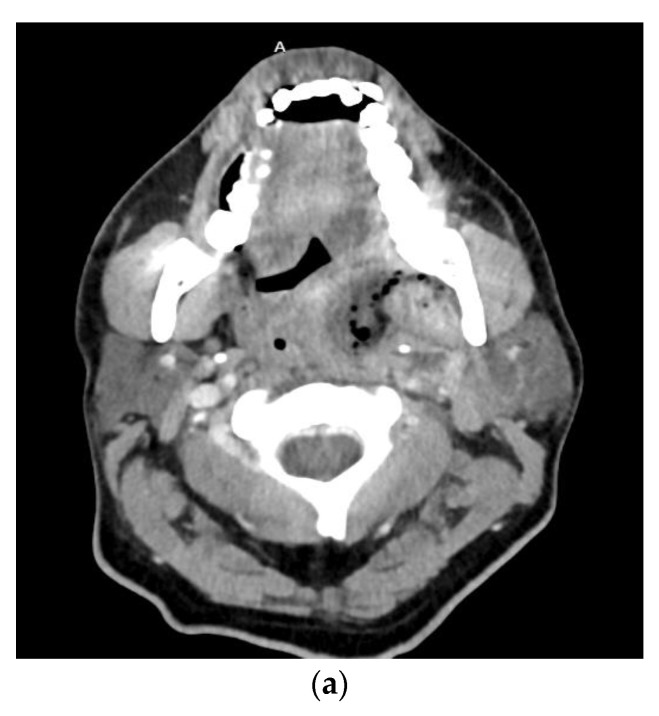

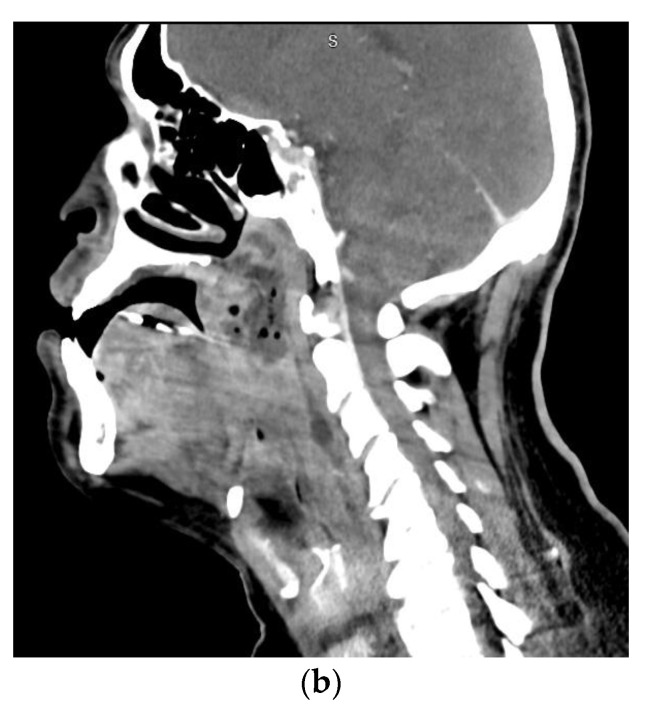
Axial (**a**) and sagittal (**b**) computed tomography scan views of a patient with a left parapharyngeal space infection with significant gas formation. Air and fluid extended within the retropharyngeal space to the level of C6/7. Bacteremia was present, with blood cultures positive for Streptococcus anginosus.

Severe odontogenic infection with progression to sepsis is rare. Given the sepsis definition update by the Sepsis-3 guidelines in 2016, it is highly unlikely a severe odontogenic infection would lead to sepsis in the absence of other complications discussed elsewhere in this article. However, bacteremia, defined as the presence of microorganisms in the blood by blood culture, may be more common Figure 3. In a study by Weise et al. of 483 patients with severe odontogenic infections, 3.3% of the study population showed a septic course. It is important to note that the criteria used to define sepsis is not described in this study. From the results, it seems the authors used the systemic inflammatory response syndrome (SIRS) criteria [20]. This criteria, as detailed by the Sepsis-3 guidelines, is useful in the general diagnosis of infection. With this in mind, the percentage of patients with septic progression from severe odontogenic infections is likely much lower. Reported risk factors that may promote septic progression in patients with severe odontogenic infections include frailty, immunosuppression, children under one year of age, adults over 75 years of age, and drug users [64]. Early detection and timely treatment of sepsis from any cause increases the likelihood of survival [65]. 

### 1.7. Necrotizing Fasciitis 

Necrotizing fasciitis is an aggressive disease that causes rapid destruction of the muscle fascia and subcutaneous tissues. The anatomic areas most commonly involved are the extremities, trunk, and perineum. While necrotizing fasciitis is rare, the mortality rate has not substantially decreased in the past 20 years, with an estimated mortality rate of 21.1% [66]. Early operative debridement and antibiotic treatment has been shown to improve mortality [67]. In a systematic review by Nawijn et al. investigating treatment timing in necrotizing soft tissue infections, patients that received surgical treatment within 6 h of presentation had a mortality rate of 19% compared to 32% in patients with surgical treatment after 6 h [66]. Prompt treatment of necrotizing fasciitis requires a high clinical suspicion, especially when patients present with underlying diabetes, immunosuppression, or liver cirrhosis. However, early recognition is difficult given the rarity of this disease and the clinical similarities to cellulitis and erysipelas. In a systematic review by Goh et al. investigating early diagnosis of necrotizing fasciitis, 71.4% of cases were misdiagnosed as cellulitis or abscess [68]. To improve early recognition, Wang et al. described three stages of necrotizing fasciitis based on cutaneous features. Stage 1 includes tenderness to palpation extending beyond the apparent area of skin involvement, erythema, swelling, and warmth. Stage 2 includes blister or bullae formation, and stage 3 includes crepitus, skin anesthesia, and skin necrosis with dusky discoloration [69]. Two clinical features that distinguish early necrotizing fasciitis from cellulitis or erysipelas are (1) poorly defined involved tissue margins; (2) tenderness extending beyond the apparent area of involvement. To further aid in distinguishing necrotizing fasciitis from other soft tissue infections, Wong et al. developed the Laboratory Risk Indicator for Necrotizing Fasciitis (LRINEC) score. The components that make up this score include C-reactive protein, white blood cell count, hemoglobin, serum sodium, serum creatinine, and blood glucose. Each component is assigned a value, and the maximum of all summed component values is 13. A score of greater than or equal to six raises clinical suspicion of necrotizing fasciitis, while a score of greater than or equal to 8 is strongly predictive of disease [70]. However, the sensitivity of this score is suboptimal at around 50% [71,72]. Computed tomography is a useful tool in detecting necrotizing fasciitis with a sensitivity greater than 80% [71,73]. The radiographic signs include fascial air or gas, soft tissue edema, or fascial enhancement. With necrotizing fasciitis, the most important factor is the time from patient presentation to surgery. The time required to obtain computed tomography imaging remains a drawback in some hospital settings. 

The incidence of necrotizing fasciitis in the United States is estimated to be 0.4 cases per 100,000 individuals [74]. While odontogenic infections are the most common cause of cervical necrotizing fasciitis, an estimated 3–10% of all necrotizing fasciitis cases involve the head and neck [75,76]. It is estimated that 1% of severe odontogenic infections progress to cervical necrotizing fasciitis [77]. Diabetes is a significant risk factor for progression with a high associated mortality. A recent systematic review of odontogenic necrotizing fasciitis reported a 9.8% mortality rate in the total study population and a 30.3% mortality rate in patients with diabetes [78]. The diagnostic value of the LRINEC score is questionable in cervical necrotizing fasciitis. In a study investigating the LRINEC score in cervical necrotizing fasciitis, Sandner et al. reported a 94% sensitivity, 94% specificity, 29% positive predictive value, and 99% negative predictive value [79]. In contrast, Thomas et al. reported a 56% sensitivity, 60% specificity, 25% positive predictive value, and 85% negative predictive value, with similar findings reported by Zemplenyi et al. [77,80]. The inclusion criteria of these studies make it difficult to analyze the clinical utility. The purpose of the LRINEC score is to identify early necrotizing fasciitis to allow for prompt treatment. The sensitivity and specificity of the LRINEC score in cervical necrotizing fasciitis cases is likely closer to that reported by Thomas et al., as this study included consecutive cases. Currently, clinical suspicion is the most important tool in the early recognition of cervical necrotizing fasciitis. 

### 1.8. Lemierre’s Syndrome

Lemierre’s Syndrome is characterized by septic thrombophlebitis of the internal jugular vein. Fusobacterium is the most common bacterial species isolated, and young healthy adults are the most common patient population affected [81]. Tonsilitis and pharyngitis are the most common causes of Lemierre’s syndrome, while just 1% of cases are from odontogenic sources. When detailed by Andre Lemierre in 1936, the mortality rate was 90% [82]. The current mortality rate has drastically decreased with antibiotic treatment to 5% [83]. The criteria historically used to diagnose Lemierre’s syndrome include (1) a primary site of infection located in the head and neck; (2) a thrombosis or thrombophlebitis of the internal jugular vein or other vein of the head and neck or metastatic lesions; (3) isolation of Fusobacterium necrophorum from blood culture or a normally sterile site [84]. This diagnostic criteria is a source of debate as cases of thrombosis or septic embolism from Fusobacterium species outside the head and neck are excluded. Additionally, this criteria does not consider other bacteria that may cause Lemierre’s syndrome [84]. The primary mechanism by which severe odontogenic infection may lead to Lemierre’s syndrome is by dissemination into the parapharyngeal space. Lemierre’s syndrome may be complicated by septic metastasis which most commonly involves the lungs and joints [85]. The clinical features typically include fever, sore throat, neck mass, and neck pain. Treatment includes antibiotic therapy and infection source control. While Lemierre’s syndrome is rare in the setting of severe odontogenic infections, it is important to recognize this syndrome given the associated morbidity and mortality.

## 2. Conclusions

The vast majority of severe odontogenic infections are routinely treated with little morbidity and mortality. With the advancement of surgical techniques, imaging modalities, and antibiotic treatments, many of these complications have become exceedingly rare. While various diagnostic tools have been developed to aid in early recognition of complications associated with severe odontogenic infections, there is no substitute for a high index of clinical suspicion. This requires an in-depth history and physical examination in conjunction with appropriate laboratory testing, imaging modalities, and appropriate multidisciplinary consultations. When underlying risk factors such as diabetes or immunosuppression are present, the clinician should be wary of the increased risk of complications. In all cases of severe odontogenic infections, patients may benefit from early antibiotic and surgical treatment. Because these complications are exceedingly rare, it is important to remember cardinal clinical features. While early treatment is important, even more so is prevention. Many patients in the United States present to the emergency department for dental related complaints and do not receive restorative or preventative treatment. All of the complications discussed in this article carry significant morbidity and mortality risks. Additionally, there are significant financial costs to the patient and the hospital system. Increased access to preventative and restorative care may decrease the hospital burden while also preventing progression to severe odontogenic infections and associated complications. 

## Figures and Tables

**Table 1:** Fascial spaces involved in severe odontogenic infections based on anatomical location with associated severity score developed by Flynn et al. [7].

Anatomic Location	Fascial Spaces Involved	Severity Score
Maxillary Teeth	Vestibular	1
	Infraorbital	1
	Buccal	1
	Infratemporal	2
Mandibular Teeth	Vestibular	1
	Buccal	1
	Submandibular	2
	Sublingual	2
	Submental	2
	Pterygomandibular	2
	Submasseteric	2
	Superficial temporal	2
Neck and Chest	Lateral pharyngeal	3
	Retropharyngeal	3
	Pretracheal	3
	Danger space	3
	Mediastinum	3

**Table 2:** The five most common bacterial species found in severe odontogenic infections based on our institution’s experience and the available literature [7,22,23,24,25].

Five Most Common Bacterial Species
Prevotella
Peptostreptococcus
Fusobacterium
Porphyromonas
Streptococcus

## Data Availability

Not applicable.
